# Pre-Clinical Efficacy and Safety of Experimental Vaccines Based on Non-Replicating Vaccinia Vectors against Yellow Fever

**DOI:** 10.1371/journal.pone.0024505

**Published:** 2011-09-09

**Authors:** Birgit Schäfer, Georg W. Holzer, Alexandra Joachimsthaler, Sogue Coulibaly, Michael Schwendinger, Brian A. Crowe, Thomas R. Kreil, P. Noel Barrett, Falko G. Falkner

**Affiliations:** 1 Department of Virology, Baxter Bioscience, Biomedical Research Center, Orth/Donau, Austria; 2 Department of Animal Housing, Baxter Bioscience, Biomedical Research Center, Orth/Donau, Austria; 3 Department of Immunology, Baxter Bioscience, Biomedical Research Center, Orth/Donau, Austria; 4 Global R&D Vaccines, Baxter Bioscience, Biomedical Research Center, Orth/Donau, Austria; Louisiana State University, United States of America

## Abstract

**Background:**

Currently existing yellow fever (YF) vaccines are based on the live attenuated yellow fever virus 17D strain (YFV-17D). Although, a good safety profile was historically attributed to the 17D vaccine, serious adverse events have been reported, making the development of a safer, more modern vaccine desirable.

**Methodology/Principal Findings:**

A gene encoding the precursor of the membrane and envelope (prME) protein of the YFV-17D strain was inserted into the non-replicating modified vaccinia virus Ankara and into the D4R-defective vaccinia virus. Candidate vaccines based on the recombinant vaccinia viruses were assessed for immunogenicity and protection in a mouse model and compared to the commercial YFV-17D vaccine. The recombinant live vaccines induced γ-interferon-secreting CD4- and functionally active CD8-T cells, and conferred full protection against lethal challenge already after a single low immunization dose of 10^5^ TCID_50_. Surprisingly, pre-existing immunity against wild-type vaccinia virus did not negatively influence protection. Unlike the classical 17D vaccine, the vaccinia virus-based vaccines did not cause mortality following intracerebral administration in mice, demonstrating better safety profiles.

**Conclusions/Significance:**

The non-replicating recombinant YF candidate live vaccines induced a broad immune response after single dose administration, were effective even in the presence of a pre-existing immunity against vaccinia virus and demonstrated an excellent safety profile in mice.

## Introduction

Yellow fever (YF) still represents a substantial threat to public health in endemic regions of tropical Africa and South America. The World Health Organization (WHO) estimated that 200,000 cases occur annually with 30,000 fatalities [1]. The causative agent of the disease, yellow fever virus (YFV), a single stranded RNA virus, belongs to the family of the *Flaviviridae* and is transmitted by mosquitoes [2]. Yellow fever disease can be divided into three stages. After an incubation period of 3–6 days, patients develop febrile illness with symptoms like fever, malaise, lower backpain, headache, myalgia, nausea, vomiting, and prostration lasting 3–4 days. Symptoms normally disappear for 2–48 hours before 15–25% of the patients enter the third phase, the period of intoxication, characterized by fever, vomiting, epigastric pain, hemorrhagic diathesis, jaundice, and liver and renal failure. Death occurs in 20–50% of severe YF cases on the seventh to tenth day [3]–[6].

As early as 1937, a live attenuated vaccine strain, yellow fever 17D, was developed based on the Asibi wild-type strain by passage in mouse and chick tissue cultures [7], [8]. The 17D vaccine has been used for many decades and has been administered to more than 400 million people [5]. The 17D vaccine, formerly classified as one of the most effective and safest available [9] is now considered to be less safe [10]. Recent studies revealed a number of vaccine related serious adverse events (SAE). Per 100,000 vaccinations 0.8 subjects developed vaccine-associated neurotropic disease [11] and 1 in 200,000 to 400,000 vaccinees developed viscerotropic disease [10]. Within the major traveler group, i.e. people over 60 years of age, the SAE incidence rate for viscerotropic disease is 1 for every 50,000 vaccinations [10]. Additionally, serious adverse outcomes, including death, have been reported in Spain, Brazil, United States, Australia, and Thailand across all age groups [12]–[18]. These reports emphasize that there is a need for a vaccine with an improved safety profile.

The YFV envelope (E) protein plays a dominant role in the induction of a protective immune response. In animal studies, purified E protein or recombinant vaccinia virus expressing prME induce high levels of neutralizing antibodies and confer immunity against lethal YFV infection [19], [20], while vaccinia recombinants expressing only non-structural YFV proteins were only partially protective [21]. Additionally, passive transfer of monoclonal anti-E antibodies demonstrated that antibody mediated immunity was sufficient to protect mice [22]. In an attempt to develop a YFV vaccine that predominantly targets the humoral immune response, an inactivated whole virus vaccine approach has recently been described [23]. However, studies indicating an important role for cellular immune responses in protection have also been published. CD4 lymphocytes bearing a Th1 phenotype in combination with antibodies were reported to play a critical role in virus clearance [24]. CD8 T cells that were induced by YFV-17D exhibited all characteristics necessary for protective cellular immunity, such as broad specificity, robust proliferation, high magnitude, and long term persistence [25], [26]. A high number of CD8- and CD4-specific T cell epitopes were mapped in the envelope protein [27]–[29].

Recombinant vaccines based on modified vaccinia virus Ankara (MVA) [30] have been used in many non clinical and clinical studies (see for instance refs. [31]–[34]). MVA has proven to be exceptionally safe [35]. No significant side effects were observed when MVA was administered to more than 120 000 humans in the context of the smallpox eradication campaign [36], [37]. Safety was also confirmed for several MVA-vectored recombinant vaccines in clinical studies [31]–[34], [38]. Due to a block in virion morphogenesis the highly attenuated vaccinia virus strain fails to productively replicate in human and most other mammalian cells [39]–[41]. Nevertheless, the ability of the virus to express viral and foreign genes in the early and late stage is retained. These characteristics make MVA a promising live vaccine vector that induces humoral and cellular immune responses and demonstrates a high safety profile. Another non-replicating vaccinia virus, the D4R defective vaccinia virus (dVV), was generated by targeted deletion of the essential vaccinia virus uracil DNA glycosylase gene (D4R), which is involved in viral DNA synthesis. Thus, in wild-type cells, the replication cycle is blocked at the stage of viral genomic replication prior to late gene expression. For propagation of dVV, an engineered cell line is used, that complements the deleted viral D4R function [42], [43]. Due to this well-defined deletion the non-replicating virus dVV represents a safe vaccine vector [44].

In this study, two new candidate vaccines were developed based on the non-replicating vaccinia viruses MVA and dVV, both expressing the YFV-17D prME open reading frame. The immunogenicity and safety of these vectors were evaluated in comparison to a commercially available YFV-17D vaccine in mice. Both candidate vaccines, induced YFV specific humoral and cellular immune responses at levels similar to the classical 17D vaccine and protected mice against a lethal YFV challenge even when pre-immunized with wild-type vaccinia virus. Notably, the vaccinia-vectored candidate vaccines showed a much better safety profile in mice than the presently used YFV-17D vaccine.

## Materials and Methods

### Ethics statement

All animal experiments were reviewed by the Baxter Bioscience Institutional Animal Care and Use Committee (IACUC Vienna/Orth) and approved by internal animal welfare officers (Experiment ID 05/07/NÖ). Animal experiments were conducted in accordance with Austrian laws on animal experimentation and approved by Austrian regulatory authorities (permit number the Government of Lower Austria, LF1-TVG-31/005-2007). Experiments were conducted according to guidelines set out by the Association for Assessment and Accreditation of Laboratory Animal Care International (AAALAC). Animals were housed according to EU guidelines, in housing facilities accredited by the AAALAC.

### Viruses and cell lines

The modified vaccinia virus Ankara [30] was obtained from the National Institutes of Health (MVA1974/NIH clone 1) and the vaccinia virus strain Lister/Elstree (VR-862) from the American Type Culture Collection (ATCC). The origin of the YFV-17D (17D) strain was the commercially available vaccine Stamaril (Sanofi/Pasteur). Primary chicken embryo cells (CEC) were generated from 12 days old chicken embryos and grown in Medium 199 (Gibco) containing 5% fetal calf serum (FCS), 100 UI/ml Pen/Strep (Lonza) and 100 UI/ml NEAA (Lonza). Vero (CCL-81), DF-1 (CCL-12203), Sol8 (CRL-2174) and HeLa (CCL-2) cell lines were obtained from the ATCC. The generation of the cell line cVero22 has been described earlier [42]. All cell lines were grown in DMEM (Biochrom) containing 5% FCS, 100 UI/ml Pen/Strep (Lonza) and 100 UI/ml NEAA (Lonza).

### Construction of plasmids

The transfer plasmid for recombination into the deletion III (del III) region of the MVA genome, was constructed in the following steps: *pd3-Script Pre1*. The left and right flanks of the del III region were amplified by PCR from genomic DNA of wild-type MVA by using the oligonucleotides oYF-8 (5′–GTT AAC AGT TTC CGG TGA ATG TGT AGA TCC AGA TAG T-3′) and oYF-9 (5′- GAA GAC GCT AGC ACT AGT GCG GCC GCT TTG GAA AGT TTT ATA GG-3′) for the right flank, and oYF-10 (5′- GCG GCC GCA CTA GTG CTA GCG TCT TCT ACC AGC CAC CGA AAG AG-3′) and oYF-11 (5′-CGT ACG TTA TTA TAT CCA TAG GAA AGG-3′) for the left flank. An overlapping PCR was performed with these two fragments as templates and the primers oYF-11 and oYF-8. The fragment was cloned into the vector pPCR-Script Amp SK (+) (Stratagene) resulting in the plasmid pd3-Script Pre1. *pd3-dlacZ/Notr-MCS*. The residual lacZ sequences and the NotI restriction site at Pos. 1617 of the pPCR-Script-Amp SK (+) plasmid were removed by BamHI, BsmFI or by BsiWI, Eel136II and mung bean digestion followed by blunt end religation resulting in the plasmid pd3-dlacZ/Notr. In order to introduce a multiple cloning site (NheI, HindIII, AluI, BamHI, StuI, SpeI, XhoI, NotI) between the vaccinia DNA segments, the plasmid was cut with NheI and NotI, and a linker consisting of the annealed oligonucleotides oYF-50 (5′- CTA GCG ACA AGC TTG CAG GAT CCA CTA GGC CTA TAA CTA GTC CGC TCG AGA TTG C-3′) and oYF-51 (5′ GGC CGC AAT CTC GAG CGG ACT AGT TAT AGG CCT AGT GGA TCC TGC AAG CTT GTC G-3′) was inserted, resulting in pd3-dlacZ/Notr-MCS. *pDW2-repeat-delIII*. A delIII self repeat (R) of the left MVA flank [45] was generated to facilitate removal of lacZ/gpt gene cassette by internal homologous recombination during plaque purification. The delIII self repeat (220 bp) was amplified by PCR from pd3-Script using the oligonucleotides oYF-48 (5′- CGC CGT CGA CTA TAT TAG ACA ATA CTA CAA TTA AC -3′) and oYF-49 (5′-ATA TGG ATC CTC TAC CAG CCA CCG AAA G-3′) and cloned between the SalI and BamHI sites of pDW2 [46] downstream of the gpt/lacZ gene cassette.

#### pd3-lacZ-gpt

The lacZ/gpt delIII self repeat fragment of pDW2-repeat-delIII was cloned into pd3-lacZ/Notr-MCS using the HindIII and BamHI restriction sites, resulting to pd3-lacZ-gpt.

#### pd3-lacZ-mH5-YFprMEco

**Figure 1:**
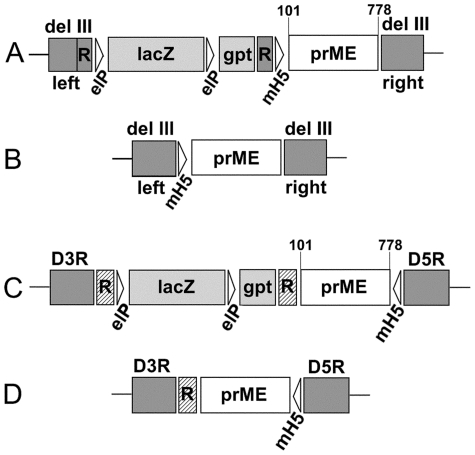
Plasmid transfer vectors (A, C) and genome structures of MVA-YF (B) and dVV-YF (D). The plasmid vector pd3-lacZ-mH5-YFprMEco (A) targets the deletion III insertion site in the MVA genome. To obtain recombinant virus (B) without any auxiliary sequences, the transient lacZ/gpt screening marker in the plasmid is flanked by a 220 bp self repeat (R) of one of the MVA flanks that mediates removal of the marker cassette by homologous recombination. The insertion site for the plasmid vector pDW-mH5-YFprMEco (C) is the region between the ORFs D3R and D5R in the wild-type Lister/Elstree virus. The lacZ/gpt marker cassette is located between tandem DNA repeats (R, hatched boxes) to achieve eventual removal of the marker cassette. The resulting recombinant defective virus (D) lacks the uracil DNA glycosylase gene (D4R), and still contains one tandem repeat [45]. Both plasmids contain the human codon-optimized YFV prM and E coding region under the control of the early/late vaccinia virus mH5 promoter.

The open reading frame encoding the YFV prME (YFprMEco) gene (Accession Number NC_002031 [47], under the control of the strong early/late vaccinia virus promoter mH5 was optimized for human codon usage (co) and synthesized (Geneart, Regensburg, Germany). The synthetic sequence is devoid of vaccinia virus early transcription stop signals; such signal was introduced immediately downstream of the coding region. The expression cassette was inserted into the SpeI/NotI site of pd3-lacZ-gpt resulting in pd3-lacZ-mH5-YFprMEco (**Fig. 1A**).

#### pDW-mH5-YFprMEco

For the generation of D4R-defective vaccinia viruses, a derivate of the plasmid pDW-2 [46] was constructed. pDW-2 contains vaccinia virus genomic sequences of the D3R and D5R genes for homologous recombination and a lacZ/gpt marker cassette located between tandem DNA repeats allowing transient selection and blue plaque screening. The synthetic mH5-YFprMEco gene cassette was inserted into the XhoI/NotI site of plasmid pDW-2 resulting in pDW-mH5-YFprMEco (**Fig. 1C**). The sequence of the promoter and prME gene cassette was verified by sequence analysis.

### Construction and purification of recombinant vaccinia viruses

#### MVA-YF (Fig. 1B)

Twenty micrograms of pd3-lacZ-mH5-YFprMEco plasmid were transfected into MVA-infected CEC by calcium phosphate precipitation. Recombinant virus was selected using the transient marker stabilization method as described previously [48], [49]. A plaque-purified clone was expanded for large scale propagation in CEC and was termed MVA-YF.

#### dVV-YF (Fig. 1D)

To generate the recombinant, replication-deficient vaccinia virus (dVV-YF) twenty micrograms of pDW2-mH5-YFprMEco were transfected into vaccinia virus Lister/Elstree infected cVero22 cells [42]. Plaque purifications were done as described earlier [43]. A plaque-purified isolate of the defective dVV-YF obtained by this procedure was amplified to large scale in cVero cells and subjected to further characterization.

### Western blot analysis

Expression of the prME protein by the MVA-YF and dVV-YF recombinants was assessed by Western blotting. To analyse the expression under permissive conditions, DF-1 cells or, in the case of the defective recombinant, cVero cells were infected with a MOI of 0.01 for 4 days. For the analysis under non-permissive conditions, HeLa or Sol8 cells were infected at a MOI of 10 for 72 h. Cells were infected in parallel with the corresponding wild-type vaccinia viruses or YFV-17D as controls. Sonicated and heat treated cell lysates were loaded on 12% polyacrylamidamide gels (Bio-Rad, Inc) and blotted onto nitrocellulose membrane (Invitrogen, Inc). To detect the prME protein, a polyclonal guinea pig (gp) antiserum against YFV-17D was used. Goat anti-guinea pig horseradish peroxidase conjugated IgG (Jackson ImmunoResearch Laboratories, Inc.) was used as a secondary antibody. YFV-17D infected HeLa cells (MOI 0.01 for 3 days) served as a positive control.

### Double immunostain assay

To detect potential contaminating wild-type virus or recombinants that lost the ability to express YF antigen, DF-1 cells or cVero22 cells were cultivated in 6 well plates and infected with 10, 100 or 1000 PFU of the recombinants. Wild-type virus and a mixture of wild-type virus and the respective recombinant were used as controls. After 1 h of incubation at 37°C in 5% CO_2_, the viral inoculum was aspirated, and 3 ml of a 0.5% carboxymethylcellulose overlay with DMEM, supplemented with 5% FCS, was added to each well. After 4 days of incubation, the overlay was removed and the cells were fixed with methanol/aceton (1∶1). To detect plaques of YFV E-protein expressors, a gp antiserum against YFV-17D was used. Goat anti-guinea pig horseradish peroxidase conjugated IgG (Jackson ImmunoResearch Laboratories, Inc.) was used as a secondary antibody. Plaques were visualized with diaminobenzidine (DAB) solutions including nickel (Vector Laboratories), resulting in black plaques. To detect MVA plaques without prME expression, a polyclonal rabbit anti-vaccinia virus serum was used (lot no. AVVSKP26012006). The secondary antibody was a goat anti-rabbit peroxidase conjugated IgG (Jackson Inc). Plaques were visualized with DAB solution without nickel, resulting in brown plaques. Black and brown plaques were counted visually.

### Immunization and challenge of animals

#### Protection

Groups of six Balb/c mice (Charles River) were immunized with MVA-YF or dVV-YF by intramuscular injections of the indicated doses in a volume of 50 µl in PBS-0.01% human serum albumin (HSA) buffer. Control groups were immunized with wild-type vaccinia viruses or YFV-17D in a volume of 50 µl in PBS-0.01% HSA or with PBS buffer. Mice were challenged intracerebrally (i.c.) with 1×10^5^ TCID_50_ (>1000 mouse lethal dose 50 (LD_50_)) of YFV-17D in TBS-0.01% HSA buffer and monitored for either 14 or 21 days for clinical symptoms and survival.

#### Passive Transfer

For generation of sera, Balb/c mice were immunized three times (d0, d21, d42) with 1×10^7^ TCID_50_ MVA-YF, dVV-YF, wild-type vaccinia virus or with 1×10^6^ TCID_50_ YFV-17D, respectively. Serum pools were prepared on day 63 and analyzed by 50% plaque reduction neutralization assay (PRNT_50_). For passive protection experiments, 8–9 weeks old mice were injected intraperitoneally (i.p.) with 150 µl undiluted or diluted sera, respectively. Six hours later, mice were challenged intracerebrally with 1×10^5^ TCID_50_ of YFV-17D and monitored for 21 days for survival.

#### Pre-existing immunity

Mice were pre-immunized intramuscularly in a single or double dose scheme with 2×10^6^ TCID_50_ of wild-type MVA or vaccinia virus strain Lister/Elstree three months before the immunization with the recombinant viruses or the controls. Immunization with the vaccinia vectored YF vaccines, and challenge was performed as described above.

#### Safety

To analyze if the integrated YF prME gene cassette alters the virulence of the recombinant vaccinia viruses, groups of 6 Balb/c mice were challenged i.c. with 1×10^5^–1×10^7^ TCID_50_ of dVV-YF, MVA-YF or the corresponding wild-type viruses. As a control, 1×10^1^–1×10^3^ TCID_50_ of the vaccine strain YFV-17D were administered intracerebrally.

### YFV plaque reduction neutralization assay (YFV-PRNT)

Approximately 3×10^5^ Vero cells were seeded per well in 6 well plates and cultured overnight to obtain confluent monolayers. Sera were complement-inactivated at 56°C for 30 min. Pre-vaccination serum was tested in 1∶10 dilution, to which 100 PFU of YFV-17D were added. Serial two-fold dilutions of the post-vaccination sera were mixed with 100 PFU of YFV-17D strain and incubated overnight at 4°C. The mixture of virus and serum were added to the Vero cell monolayers and incubated for 1 hour at 37°C. Virus/serum mixtures were replaced by 0.75% carboxymethylcellulose-DMEM solution, incubated for 4 days and visualized by immunostaining as described above. The neutralizing antibody titer is the reciprocal of the highest serum dilution that reduced the number of viral plaques by at least 50% relative to the pre-vaccination sera.

### Vaccinia plaque reduction neutralization assay (VV-PRNT)

The test for neutralizing antibodies against vaccinia virus was performed as described above, with the difference that vaccinia virus strain Lister/Elstree (ATCC VR-862) was used as the target virus, and neutralization was done at 37°C for 1 hour. VV plaques were stained with crystal violet.

### Detection of antigen-specific T cells in immunized mice

Five Balb/c mice per group were immunized as described above, spleens were collected at day 28 post-immunization, and pooled splenocyte cell suspensions were prepared. Vaccine-specific cell-mediated immunity was evaluated as described previously [42] using flow cytometric interferon-γ (IFN-γ) response assays and analysis of killing of peptide-pulsed target cells by specific CD8 T cells (VITAL assay). For the intracellular cytokine staining assay, splenocytes were restimulated using 2 µg/ml of the following previously described [29] synthetic peptides from the yellow fever envelope protein: E57–71, E129–143, E133–147 (15mer peptides recognized by CD4 T cells) and E60–68, E330–338, E332–340 (9mer peptides recognized by CD8 T cells). As negative controls, cells were also stimulated with 15mer and 9mer peptides from influenza haemagglutinin, QTKLYQNPTTYISVG and IYSTVASSL, respectively. Medium control background frequencies were subtracted. In the VITAL assay, 5-(and-6)-carboxyfluorescein diacetate succimidyl ester (CSFE)-stained target cells were loaded with the 9mer peptides E60–68, E330–338, E332–340 at 2 µM, while 5-(and-6)-(((4-chloromethyl)benzoyl)amino) tetramethylrhodamine (CMTMR)-labeled control cells were pulsed with the H2-K^d^ -restricted peptide IYSTVASSL from influenza hemagglutinin.

### Statistical analysis

Data were analyzed for statistical difference by performing one-way analysis of variance (ANOVA, Tukey-Kramer multiple comparisons test) using the GraphPad Prism software (San Diego, CA). The differences were considered significant when p<0.05.

## Results

### Construction and Characterization of the Recombinant Virus MVA-YF

We constructed a recombinant MVA, designated MVA-YF, that expresses the prME coding sequence (CDS) of yellow fever virus strain 17D. The prME CDS under the control of the vaccinia virus early/late mH5 promoter [50] was chemically synthesized. This allowed the removal of poxvirus early transcription termination signals (5TNT) present in the original sequence and the optimization of the open reading frame for human codon usage to achieve maximal expression levels in humans without modifying the amino acid sequence.

To generate MVA-YF, the codon-optimized (co) expression cassette was inserted into the newly constructed transfer plasmid pd3-lacZ-gpt resulting in the plasmid pd3-lacZ-mH5-YFprMEco (**Fig. 1A**). This plasmid directs the foreign gene into the deletion III region of MVA by homologous recombination. After several rounds of plaque purification, initially with, then without, selective pressure [48] the final recombinant virus, designated MVA-YF, was obtained (**Fig. 1B**). This virus contains the prME gene regulated by the vaccinia virus mH5 promoter in the MVA del III insertion site and is free of additional foreign sequences.

**Figure 2:**
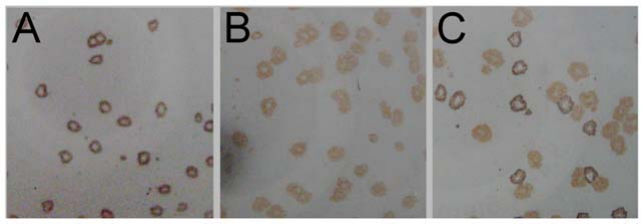
Double Immunostaining of infected chicken cells (DF-1). MVA-YF (A), wild-type MVA (B) and MVA-YF/MVA spike control (C). After 4 days infected cells were fixed, incubated with guinea pig anti YFV-17D antiserum and anti-guinea pig IgG conjugated to peroxidase. Expressors of prME were visualized as black plaques staining with DAB solution with nickel. To detect MVA without prME expression, cells were incubated with rabbit-anti-vaccinia virus serum and anti-rabbit peroxidase-conjugated IgG antibody and subsequent staining with DAB solution without nickel, resulting in brown plaques (prME non-expressors).

The absence of wild-type MVA from the recombinant virus was checked by PCR analysis. This assay confirmed that the recombinant MVA-YF stock was free from parental wild-type virus at a detection limit of about 1 plaque forming unit (PFU) contaminants among 1000 PFU of recombinant virus (data not shown). To confirm the absence of wild-type MVA on the phenotype level, a double immunostain assay was performed. For this purpose, DF-1 cells were infected with MVA-YF, wild-type MVA or with a 1∶1 mixture of both viruses as a control. After incubation for four days, cells were fixed and stained with an anti-YFV- followed by an anti-VV- antiserum, resulting in black and brown plaques, respectively. The MVA-YF infected cells showed uniformly black foci representing recombinants expressing prME proteins (**Fig. 2A**), which indicated that the stock was free from wild-type MVA or aberrant recombinants without prME expression (non-expressors). In the wild-type MVA control only brown foci of non-expressors were seen (**Fig. 2B**), whereas the MVA-YF/MVA spike control contained clearly distinguishable brown and black foci in the expected ratio (**Fig. 2C**).

### Construction and Characterization of the Recombinant Virus dVV-YF

In parallel, a D4R-defective vaccinia virus (dVV) expressing the codon-optimized prME CDS was generated. For this purpose, the mH5-prME cassette was inserted into the plasmid pDW2 resulting in the transfer plasmid pDW-mH5-YFprMEco (**Fig. 1C**). This plasmid was used to construct the non-replicating virus dVV-YF, in which the YFV prME expression cassette is inserted between the vaccinia D3R and D5R genes, knocking out the essential D4R gene. Recombinant virus was generated by infecting D4R-complementing cVero22 cells with wild-type VV (strain Lister/Elstree), and by transfection of the recombination plasmid and several rounds of plaque purification as described in the methods section. These steps resulted in the replication-deficient recombinant virus, termed dVV-YF (**Fig. 1D**). The recombinant had the intended genetic structure without any marker gene, as characterized by PCR. It was growth-defective in wild-type cells, and all plaques analyzed by double immunostaining expressed prME proteins (data not shown).

### Antigen Expression in Permissive- and Non Permissive Cells

**Figure 3:**
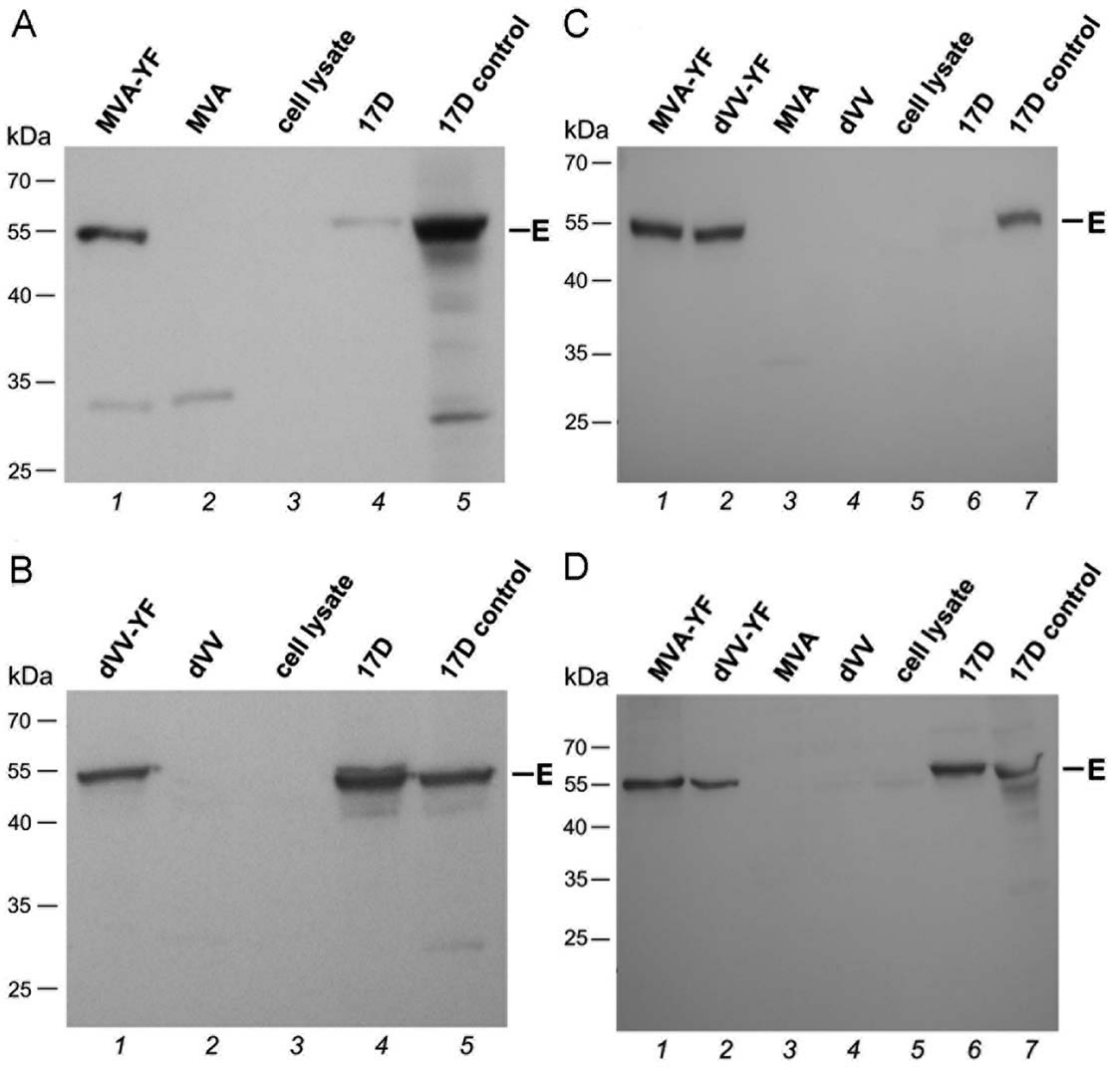
YFV prME protein expression under permissive (A and B) and non-permissive (C and D) conditions. (A) Western blot of lysates from chicken cells (DF-1) infected with MVA-YF or the corresponding controls. MVA-YF (Lane 1), wild-type MVA (Lane 2), non-infected DF-1 cells (Lane 3), positive control YFV-17D infected DF-1 cells (17D, Lane 4), YFV-17D prepared from infected HeLa cells (17D control, Lane 5). (B) Western blot of lysates from cVero22 cells infected with dVV-YF or the corresponding controls. dVV-YF (Lane 1), wild-type dVV (Lane 2), non-infected cVero22 cells (Lane 3), positive control YFV-17D infected cVero22 (17D, Lane 4), 17D control (Lane 5). Western blot of mouse muscle cells (Sol8, C) or human cells (HeLa, D) infected with the recombinants or the corresponding controls. MVA-YF (Lane 1), dVV-YF (Lane 2), wild-type MVA (Lane 3), wild-type dVV (Lane 4), non-infected Sol8 (C) HeLa (D) cells (Lane 5), cells infected with YFV-17D (17D, Lane 6), 17D control (Lane 7). The band around 55 kDa marked “E” indicates the YFV envelope protein.

The prME expression pattern by MVA-YF was first tested under conditions that are permissive for MVA replication. For this purpose, avian DF-1 cells were infected with a MVA-YF or with wild-type MVA or YFV-17D as controls at a MOI of 0.01. Infected cells were incubated for four days and total cell lysates were investigated by SDS-PAGE and Western blot analysis using polyclonal anti-YFV-17D antiserum. As shown in **Fig. 3A**, the YF envelope (E) protein expressed by the recombinant MVA-YF (lane 1) appeared as a single band in the 55 kDa size range, which is the expected size of flavivirus E proteins [2]. An identical band was also detectable in the YFV-17D infection (lane 4). The E protein expression level of the recombinant MVA was higher than in the YFV-17D infection. The low expression level of YFV-17D in avian DF-1 cells was seen repeatedly.

To investigate the prME expression by dVV-YF in a cell culture system that supports replication of D4R defective vaccinia, the complementing Vero cell line cVero22 was infected with a MOI of 0.01 with dVV-YF or with the dVV wild-type virus or YFV-17D as controls. Further steps were performed as described above. As shown in **Fig. 3B** the recombinant dVV-YF (lane 1) expressed the E protein in the infected cVero22 cells, as did the YFV-17D virus (lane 4).

The recombinant viruses MVA-YF and dVV-YF were designed as live vaccines for human use, and efficacy was assessed in a mouse protection model. In the mouse and human organisms, these vaccinia viruses do not replicate. Efficient YFV prME expression also without replication of the vaccinia vector is therefore a prerequisite for successful immunization. For this reason, the expression patterns were also studied in a human and in a mouse cell line, non permissive for both the recombinant MVA-YF and dVV-YF. Mouse muscle (Sol8) or human (HeLa) cells were infected with a MOI of 10 of MVA-YF or dVV-YF and with the corresponding controls. Infected cells were incubated for two days and total cell lysates were investigated by SDS-PAGE and Western blot analysis using anti-YFV antiserum. The expression in Sol8 muscle cells should reflect the target cell type in the selected mouse challenge model in which mice are immunized intramuscularly (i.m.). As shown in **Fig. 3C**, recombinant MVA-YF (lane 1) and dVV-YF (lane 2) expressed the E-protein in comparable amounts. As expected, in the negative controls (lanes 3–5) no YFV protein was detectable. In this setting, E-protein expression by the YFV-17D positive control (lane 6) was below the limit of detection. In HeLa cells representing the human situation (**Fig. 3D**) again comparable amounts of E-protein were found in MVA-YF (lane 1) and dVV-YF (lane 2) infections. The YFV-17D infected cells (lane 6) yielded similar amounts of E-protein. Thus, in humans vaccinated with non-replicating MVA-YF or dVV-YF, correct expression of the YFV antigen at significant levels can be expected.

### Protection Studies in Mice

The capacity of recombinant MVA-YF and dVV-YF to protect mice against a lethal i.c. challenge with YFV-17D virus was analyzed. Balb/c mice were therefore immunized with a single intramuscular injection of MVA-YF or dVV-YF over a dose range of 1×10^2^ to 1×10^5^ tissue culture infectious dose 50% (TCID_50_). Additionally, mice were immunized with 1×10^6^ TCID_50_ of wild-type MVA, dVV or PBS as negative controls, and with 1×10^4^ TCID_50_ (equivalent to the human dose) of YFV-17D as positive control. Mice were challenged i.c. with 1×10^5^ TCID_50_ per animal, representing a more than 1000-fold LD_50_ of the vaccine strain YFV-17D at day 21 post vaccination. The LD_50_ in nine week old mice was determined to be approximately 83 TCID_50_ (data not shown).

**Figure 4:**
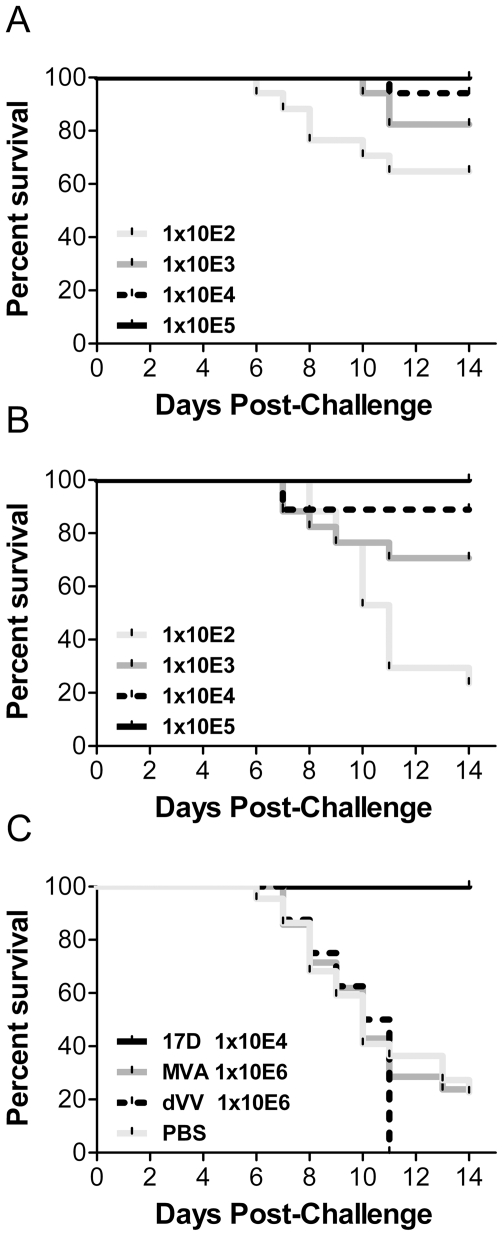
Protection studies in Balb/c mice. Animals were vaccinated i.m. in a single dose scheme with the indicated doses of (A) MVA-YF, (B) dVV-YF or with (C) the positive control YFV-17D (17D) and the negative controls wild-type MVA, defective vaccinia virus (dVV) or buffer (PBS). Mice were challenged i.c. 21 days later with 1×10^5^ TCID_50_ of YFV-17D vaccine strain and monitored for 14 days. Results are the average of 3 individual experiments.

Protection was demonstrated to be clearly dependent on the dose of the vaccinia-vectored vaccines, **Fig. 4A** shows the results obtained with MVA-YF while **Fig. 4B** shows those of dVV-YF. The highest dose of 1×10^5^ TCID_50_ per animal conferred full protection. The overall protection by dVV-YF was somewhat weaker than by the recombinant MVA-YF. At the dose of 1×10^2^ TCID_50_ the survival with MVA-YF (65%±11.6) was significantly (p<0.01) higher than with dVV-YF (24%±10.3). All negative control groups, injected with the wild-type vaccinia viruses or PBS, showed survival rates of maximal 20%. As expected, complete protection was also seen in groups immunized with YFV-17D (**Fig. 4C**).

Neutralizing antibodies were analyzed on day 19 in pre-challenge sera. After vaccination with a single dose, the PRNT_50_ titer was low. This was true for vaccinations with recombinant viruses up to the highest and fully protective immunization doses, but also with YFV-17D. Therefore, a further experiment was performed in which mice received single or double dose inoculations of 10^4^, 10^6^ and 10^7^ TCID_50_ of MVA-YF or dVV-YF or 10^4^ and 10^6^ TCID_50_ of YFV-17D virus as a positive control. Additionally, mice were immunized with a double dose of 1×10^7^ TCID_50_ of the wild-type MVA or dVV vectors as negative controls. Sera were collected at day 42 after the primary immunization and analyzed for YFV neutralizing antibodies by PRNT_50_ assay. Again, both recombinant vaccines induced 100% protection after one application of 10^5^ TCID_50_ (**Table 1**), however no or only low PRNT_50_ titers were measureable even at the highest administered dose of 10^7^ TCID_50_. Only the YFV-17D vaccine this time induced low, but measurable neutralization titers after a single dose administration of 10^4^ and 10^6^ TCID_50_.

**Table 1:** Protection and pre-challenge YFV plaque reduction neutralization titers in mice.

Vaccine	Immun. Dose [log10]	Protection [Survivors/Total (%)](3)	PRNT_50_ [GMT](1 ^,^ 2)	PRNT_50_ [GMT](1 ^,^ 2)
		Single Dose	Single Dose	Double Dose
MVA-YF	2	11/17 (65)	<10(6)	n.d.
	3	14/17 (82)	<10(6)	n.d.
	4	16/17 (94)	<10	17
	5	17/17 (100)	<10(6)	n.d.
	6	n.d.(4)	<10	42
	7	n.d.	<10	80
dVV-YF	2	4/17 (24)	<10(6)	n.d.
	3	12/17 (71)	<10(6)	n.d.
	4	16/18 (89)	<10	18.2
	5	18/18 (100)	<10(6)	n.d.
	6	n.d.	17.3	26.2
	7	n.d.	<10	33
17D	4	17/17 (100)	13.2	120
	6	-	54.3	381
MVA	6/7(5)	5/21 (24)	<10	<10
dVV	6/7(5)	8/8 (0)	<10	<10
Buffer	-	5/22 (23)	<10	<10

(1) Geometric mean titer;

(2) Results of two independent experiments;

(3) results of three independent experiments (except dVV);

(4) not determined;

(5) protection studies 10^6^ TCID_50_, PRNT studies 10^7^ TCID_50_;

(6) determined at d19.

After a second vaccination with MVA-YF or dVV-YF, neutralization titers were detectable in a dose-dependent fashion. Here, the MVA-based vaccine showed on average somewhat higher titers than the dVV-YF vaccine. The highest neutralization titers were induced with the YFV-17D vaccine.

### Protection of Balb/c mice by passive transfer of immune sera

In the immunization experiments, full protection was achieved with the recombinant vaccines at neutralizing antibody levels below the limit of detection. To independently analyze the contribution of neutralizing antibodies to protection high titer sera were produced. Balb/c mice were immunized three times intramuscularly with 10^7^ TCID_50_ of MVA-YF, dVV-YF and YFV-17D. In parallel, mice were immunized i.m. with a triple dose of 10^7^ TCID_50_ of the wild-type MVA or dVV vectors as negative controls. These high i.m. injections did not cause any signs of disease in the mice confirming that the vaccines are well tolerated using this route. Sera were collected on day 63 after the primary immunization and analyzed by PRNT_50_ assay.

Groups of six 8–9 weeks old mice were then injected intraperitoneal (i.p.) with 150 µl of sera with the indicated PRNT_50_ values (**Table 2**). Six hours after passive transfer these groups and a control non-treated group were challenged i.c. with 10^5^ TCID_50_ of YFV-17D. The data presented in **Table 2** demonstrate that the mean survival time of treated and non-treated groups were similar and passively transferred antibodies induced only partial protection from lethal challenge. In this model at least seventy to eighty percent of mice that received sera of high titers (PRNT_50_ 50–100) from MVA-YF immunized mice survived, whereas all mice that received sera with the low titer (PRNT_50_ 10) died. Mice treated with high titer sera of dVV-YF (PRNT_50_ 70) and YFV-17D (PRNT_50_ 100) immunized mice showed survival rates of 27% and 25%, respectively. Low titer treated mice (PRNT_50_ 5–7) showed similar survival rates as control groups. Differences between the performance of the sera from the individual vaccines were not significant. In conclusion, a relatively good passive protection level of 70–80% was seen after passive transfer with the MVA-YF-induced sera confirming the important role of neutralizing antibodies in protection, however, suggesting that full protection in the active immunizations is supported by the T cell responses in this animal model.

**Table 2:** Protection and mean survival time after passive transfer.

Vaccine	Passive Transfer Sera [PRNT_50_]	Mean survival time [Days±SEM](1)	Protection [Survivors/Total (%)](1)
MVA-YF	100	10.3±2.33	8/11 (73)
MVA-YF	50	11.0±1.00	9/11 (82)
MVA-YF	10	9.1±0.48	0/12 (0)
dVV-YF	70	9.8±1.03	3/11 (27)
dVV-YF	7	9.4±0.61	1/12 (8)
17D	100	9.8±0.83	3/12 (25)
17D	50	10.1±0.64	2/12 (17)
17D	5	10.8±0.58	0/12 (0)
MVA	<10	8.5±0.58	0/12 (0)
dVV	<10	9.7±0.48	0/12 (0)
-	<10	8.5±0.47	1/12 (8)

(1) results of two independent experiments.

### Induction of envelope protein-specific T cell responses in mice

While induction of a humoral immune response and generation of neutralizing antibodies against the envelope protein represent the major protective mechanism following vaccination with the live YFV-17D vaccine [6], [51], the cellular immune responses are also thought to play an important role in protection against infection [24], [27]–[29]. Recently, the T cell responses induced by the YFV-17D vaccine were characterized [29]. In the latter study, BALB/c (H2d) mice were inoculated with the 17D vaccine strain and CD8- and CD4-specific epitopes were investigated [29].

**Figure 5:**
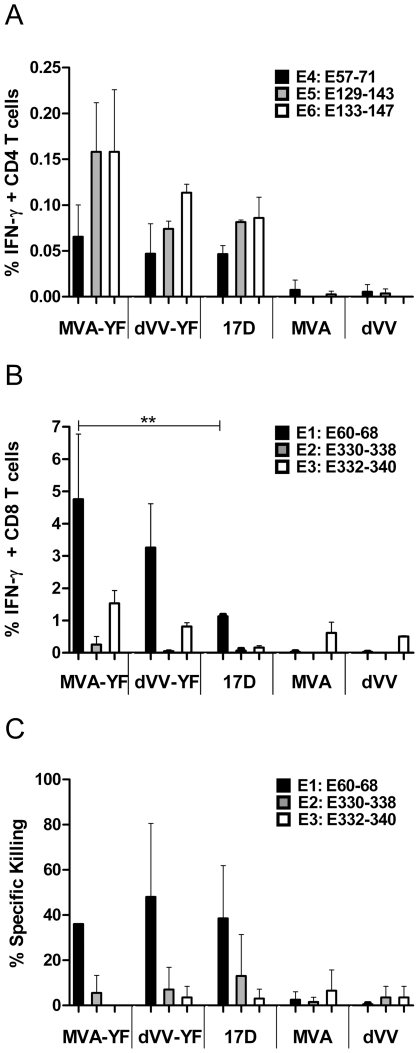
Cellular immune response elicited against YFV E-antigen. (A) FACS analysis of the number of IFN-γ secreting CD4^+^ T-cells after two immunizations with MVA-YF, dVV-YF or the corresponding YFV-17D (17D) positive or wild-type MVA and dVV negative controls. Splenocytes from mice were stimulated with 15mer peptides of the YFV E-protein, E57–71 (E4; black bars), E129–143 (E5; grey bars) and E133–147 (E6; white bars). (B) FACS analysis of the number of IFN-γ secreting CD8^+^ T cells after the two immunizations as indicated above. Splenocytes from mice were stimulated with 9-mer peptides of the YFV E-protein, E60–68 (E1; black bars), E330–338 (E2; grey bars), E332–340 (E3 white bars); ** p<0.001. (C) FACS analysis of cytotoxic killing of peptide-pulsed target cells by specific CD8^+^ T cells. Target cells were loaded with 9mer peptides of the YFV E-protein, E60–68 (E1; black bars), E330–338 (E2; grey bars), E332–340 (E3 white bars). The data are mean values (+/− SD) of two independent experiments.

To compare the T-cell responses following MVA-YF or dVV-YF vaccination to the YFV-17D vaccine, mice were immunized twice (day 0 and day 21) with the vaccinia virus recombinants or the corresponding controls. Splenocytes were prepared on day 28 and stimulated in vitro with CD8- and CD4-specific peptides derived from the YFV envelope [29]. The percentages of IFN-γ producing T cells were determined by a FACS-based intracellular cytokine assay. The results of CD4-specific responses of two independent experiments obtained with the prME expressing recombinants MVA-YF, dVV-YF and with the corresponding controls are shown in **Fig. 5A**. Following stimulation with YFV E-peptides E4-E6, the vectors dVV-YF and YFV-17D induced comparable amounts of specific CD4 T cells. The highest frequency of CD4 positive IFN-γ producing T cells (0.16%) was seen after recombinant MVA-YF immunization, the difference to YFV-17D was however not significant (p>0.05). Mean responses to the 15mer control peptide from influenza haemagglutinin were <0.01% in all vaccination groups (data not shown).

The frequency of YFV-specific CD8 T cells induced by the recombinants and controls upon in vitro stimulation with the YFV E-peptides are shown in **Fig. 5B**. Up to 5% of the total CD8 T cells responded to the immunodominant peptide E1. The highest frequency of YFV-specific CD8 T cells were detected in the mice immunized with the MVA recombinant, followed by dVV-YF. CD8 T cell activation by the recombinants was higher than by the YFV-17D vaccine. This difference was significant for MVA-YF (p<0.001). Again, responses to the 9mer control peptide from influenza were low in all groups (<0.1%, data not shown).

To verify that the envelope-specific CD8 T cells were functionally active and kill target cells pulsed with specific YFV envelope peptides, a cytotoxic T-lymphocyte (CTL) killing assay based on fluorometric techniques was used [52]. For this purpose, splenocytes were incubated with peptide-presenting and dye-labeled target and control cells. The reduction of the peptide-pulsed target cells versus control cells after incubation with the splenocytes indicates the presence of functional CTLs. As already seen in the CD8 IFN-γ assay, pulsing with E1 resulted in the highest responses (**Fig. 5C**). CTL-specific killing was comparable for the groups immunized with MVA-YF (36%±0), dVV-YF (48%±23) and YFV-17D (38.5%±16.5). In summary, immunization with the MVA and dVV recombinants and with YFV-17D vaccine induced comparable levels of functionally competent CTLs.

### Influence of pre-existing immunity on protection

It can be assumed that a subset of the human population possesses immunity to vaccinia virus due to previous vaccinations, either having received smallpox vaccination or being vaccinated with MVA recombinant vaccines. Thus it is important to analyze the influence of an already existing immunity to the vector on the protection by the recombinant vaccine. To investigate if previous exposure to vaccinia virus influences the effectiveness of the recombinants, Balb/c mice were immunized first with 2×10^6^ TCID_50_ wild-type MVA (single and double dose) or vaccinia virus Lister/Elstree, respectively. Three months later, animals were vaccinated with a suboptimal double dose of 1×10^3^ TCID_50_ or with the usually protective single dose of 1×10^5^ TCID_50_ of MVA-YF, and the corresponding controls. Animals were finally challenged with more than a 1000-fold LD_50_ YFV-17D. The design of the experiment and the results are outlined in **Table 3**. Prior to immunization with MVA-YF, sera were collected to determine vaccinia virus-specific neutralizing antibody titers (PRNT_50_).

**Table 3:** Immunization scheme, VV PRNT_50_ values prior to YF vaccination and survival of mice.

group	1st pre-Immun.	2nd pre-Immun.	VV PRNT_50_ [GMT](1)	1st Immun.	2nd Immun.	protection [Survivors/Total (%)]
	[day 0]	[day 21]	[day 82]	[day 84]	[day 104]	[day 138]
1(3)	VV-Lister	-	57(1)	MVA-YF (10^3^)	MVA-YF (10^3^)	5/9 (56)
2(2)	MVA		403(1)	MVA-YF (10^3^)	MVA-YF (10^3^)	9/16 (56)
3(3)	MVA	MVA	453(1)	MVA-YF (10^3^)	MVA-YF (10^3^)	4/11 (36)
4(2)	-	-	<20	MVA-YF (10^3^)	MVA-YF (10^3^)	9/16 (56)
5(3)	MVA	MVA	392(1)	MVA-YF (10^5^)	-	8/11 (73)
6(3)	-	-	<20	MVA-YF (10^5^)	-	10/11 (91)
7(2)	-	-	<20	17D (10^4^)	-	13/18 (72)
8(2)	-	-	<20	Buffer	-	1/16 (6)

(1) Geometric mean titer;

(2) results of three independent experiments;

(3) results of two independent experiments.

In the groups immunized with a suboptimal double dose of MVA-YF, no significant effect of the different pre-vaccinations with wild-type viruses was seen (**Tab. 3**, groups 1–4). The difference between protection achieved without (56% survival) or with pre-existing immunity by wild-type VV immunization (VV PRNT_50_ 57–453, survival 36–56%) was not significant in all cases (p>0.05).

Almost all mice (91%) which obtained a usually protective single dose of 1×10^5^ TCID_50_ MVA-YF (**Tab. 3**, group 6) survived after challenge if no pre-vaccination was performed. Also in this case, pre-vaccination with a double dose of wild-type MVA (group 5) did not result in significantly (p>0.05) decreased protection. In summary, pre-existing anti-vaccinia virus immunity had no significant negative influence on the protection of MVA-YF against a lethal YFV-17D challenge.

### Safety of MVA-YF and dVV-YF

**Figure 6:**
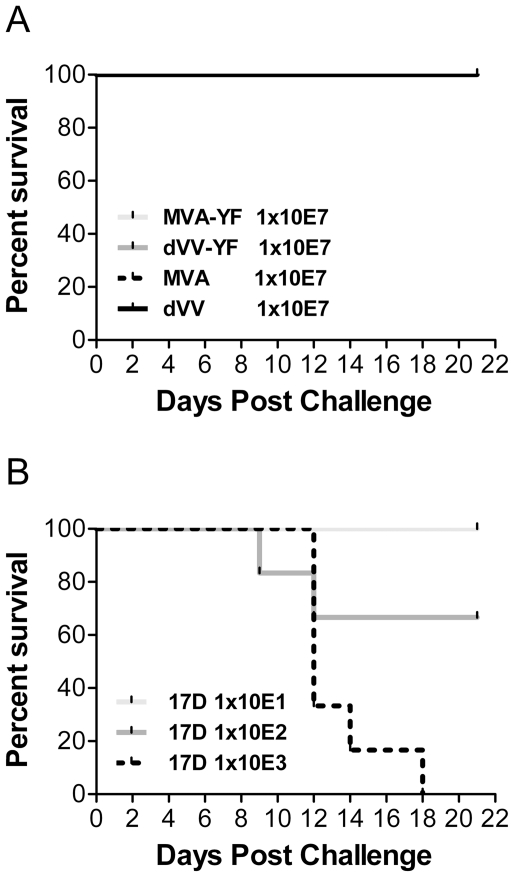
Safety of recombinant candidate vaccines in BALB/c mice. (A) Animals were injected i.c. with 1×10^5^ to 1×10^7^ TCID_50_ (only 1×10^7^ TCID_50_ dose shown) of MVA-YF (bright grey line), dVV-YF (grey line) and the corresponding controls wild-type MVA (dotted line) and dVV (black line) and monitored for 21 days. (B) Mice were injected i.c. with YFV-17D vaccine at doses of 1×10^1^ (bright grey line), 1×10^2^ (grey line) or 1×10^3^ (dotted line), and monitored for 21 days.

To exclude the possibility that the introduction of the YFV prME gene might alter the infectivity of the vaccinia virus vectors, the safety profiles of the vaccines were tested. For this purpose, Balb/c mice were challenged i.c. with high doses of 1×10^5^ to 1×10^7^ TCID_50_ MVA-YF, dVV-YF and with the corresponding wild-type viruses. To compare also the safety profile of the recombinants with the YFV-17D vaccine, 1×10^1^ to 1×10^3^ TCID_50_ of YFV-17D were administered intracerebrally. In the vaccinia virus challenged groups, complete survival was seen even with the highest dose of 1×10^7^ TCID_50_ (**Fig. 6 A**). Furthermore, there was no difference between the wild-type vaccinia vectors and the recombinants. In contrast, in YFV-17D challenged mice, a very low dose of 1×10^2^ TCID_50_ YFV-17D induced 65% lethality and 1×10^3^ TCID_50_ killed 100% of the mice (**Fig. 6 B**). In conclusion the non-replicating vaccinia-based vaccines were safe and very high doses administered intracerebrally did not kill the mice. Furthermore, the introduction of the prME gene did not altered the safety profile of the vaccinia vectors while low doses of the YFV-17D vaccine as expected killed the mice after i.c. administration.

## Discussion

In order to combine the advantages of the existing YF live vaccines with the excellent safety profile of MVA, we generated a recombinant YF vaccine on the basis of this vector. In parallel, we investigated an analogous vaccine on the basis of dVV, a vaccinia virus that is rendered non-replicating by genetic engineering. This virus retains its infectivity and expresses viral and integrated foreign genes but fails to complete the replication cycle. The virus can only be propagated to normal titers in an engineered complementing cell line, but it is replication-deficient in any natural host. Thus, dVV can be considered as a safe live vaccine candidate similar to MVA [44]. The recombinant vaccines were constructed by use of a synthetic sequence encoding the structural proteins prME of the attenuated YFV-17D strain [47], optimized for human codon-usage. The prME of the 17D strain was chosen because the antigen is highly protective as proven by vaccination campaigns in the past decades. Furthermore, use of identical antigens ensures a valid comparison of the new VV based vaccine concepts with the commercial YFV-17D live vaccine.

In the present study, we could show full protection of Balb/c mice against a highly lethal challenge with YFV-17D (>1000-fold mouse LD_50_) by the vaccinia-vectored vaccines. Surprisingly, relatively low doses of 1×10^5^ TCID_50_ of recombinant MVA or dVV, respectively, were sufficient to confer full protection after only one vaccination. In a previous study reported by another group [20], a single dose of 1×10^7^ PFU of the replication competent vaccinia virus Western Reserve expressing the YFV-17D prME could only partially protect mice against a 100-fold LD_50_ challenge. Even after two inoculations, only 94% survival of the animals was reported. In that study, however, the French neurotropic YFV strain was used for challenge. Although in the previous study, the LD_50_ dose was 10-fold lower, the use of the French neurotropic as heterologous challenge strain, and the different tropism of the viruses might have contributed to the divergent protection results reported here. Nevertheless, the superior immunogenicity achieved with MVA vectors compared to replicating vaccinia virus Western Reserve strain [53] and use of codon-optimized gene cassettes driven by a strong promoter might explain the improved protection in the present study. In studies with recombinant MVA expressing the Japanese encephalitis virus (JEV) prME genes, three doses of 2×10^6^ infectious units were necessary to protect mice against JEV challenge [54]. However, in that case challenge was extremely high. Mice were challenged with 100 000 LD_50_, a dose that was 100-fold higher than in our study.

MVA-based vaccines have been used in clinical studies, for instance, against HIV [32], tuberculosis [31], malaria [33] and cancer [34]. In all of these studies, at least two doses were used. The human dose of an MVA-based vaccine was 5×10^7^ to 5×10^8^ PFU as applied in recent clinical trials [31]–[34], [55]. From the results of our present animal studies, we speculate that the MVA-YF vaccine might be efficient in a single dose regimen, at comparable doses. It would offer significant cost and logistical benefits, if MVA-YF could be administered in a single dose scheme.

The induction of YFV-specific neutralizing antibodies in Balb/c mice after a single dose immunization with MVA-YF and dVV-YF was moderate. The PRNT_50_ titers at days 19 or 42, respectively, were below or close to the limit of detection, even after the administration of 10^7^ TCID_50_. At the same time, a single dose of 10^5^ TCID_50_ already induced 100% protection. Only after two vaccinations were dose-dependent neutralization titers detectable. These findings differ from previous results, where a single dose of 10^7^ PFU of replicating recombinant vaccinia virus induced measurable neutralizing antibodies to YFV in CD-1 mice [20]. However, in that study, also the YFV-17D control induced higher neutralization titers than observed in our study. These differences may be due to different mouse strains used or to assay specific parameters. A head-to-head comparison of Balb/c and CD1 mice was conducted in immunization experiments with inactivated whole YFV-17D candidate vaccine [23]. In our study, two doses of 10^6^ TCID_50_ MVA-YF resulted in similar neutralizing antibody titers in Balb/c mice as reported for a proposed human dose of the inactivated candidate vaccine [23]. Interestingly, with the inactivated vaccine, higher antibody levels were published in CD1 mice. Since the inactivated candidate vaccine was tested in the mouse model only for immunogenicity but not for protective efficacy, a comparison of the protective antibody levels between the inactivated and the vaccinia vectored live vaccine is not possible.

To explore the ability of neutralizing antibodies to account for protection of mice from lethal challenge, passive transfer of serum from vaccinated mice to naïve mice were performed. Mice which received low titer sera and sera of wild-type MVA or dVV immunized mice died or showed only background survival rates. Partial protection of mice was only achieved after passive transfer of sera from MVA-YF, dVV-YF and YFV-17D immunized mice with PRNT_50_ of about 100. This corresponds to titers of approximately 10 in the bloodstream of animals, assuming that the transferred serum is diluted approximately tenfold in mouse blood volume [56]. In the active immunization model full protection was conferred after vaccination with 1×10^5^ TCID_50_ of the recombinant candidate vaccines. At the same time the neutralizing antibody titer was below the technical detection limit. Based on the known role of neutralizing antibodies in flavivirus protection, we assume that neutralizing antibodies below our detection limit of <10 were present. However, these data further suggest that additional factors, such as the cellular immune response contributes to protection.

The induction of the E-protein specific cellular immune responses is considered to be an important factor of immunity for the live YFV-17D vaccine. We present for the first time an analysis of the cellular immunity to E-antigen induced by recombinant non-replicating vaccinia viruses and a comparison with the YFV-17D live vaccine. Both vaccinia virus-based vaccines induced a strong cellular immune response eliciting particularly high frequencies of antigen-specific CD8-T cells but induction of antigen-specific IFN-γ producing CD4 T cells could also be demonstrated. Specific CD4 T cell levels were higher with the MVA-YF vaccine than with the dVV-YF or YFV-17D vaccines, which showed comparable responses.

Administration of the MVA-based vaccine also resulted in the strongest induction of E-antigen specific IFN-γ secreting CD8 T cells followed by the dVV-based and YFV-17D vaccines. However, comparable amounts of E protein-specific CTLs were induced by all three vaccines. In a previous study, no deleterious effects on the protection following YFV-17D immunization were seen when CD8 knock-out mice were used [24]. This suggested that CD8 T cells do not represent a critical component of the protection against lethal encephalitis in mice. Nevertheless, CD8 T cells may contribute to virus clearance in humans who develop viscerotropic disease. A recent study analyzed the attributes of human CD8 T cell response after YFV-17D vaccinination in humans and found broad specificity, multiple function, robust proliferation, and long term persistence, all characteristics of protective cellular immunity [25].

In our studies, surprisingly low doses of the vaccinia vectored vaccines conferred protection at least as good as a full human dose of the YFV-17D live vaccine. A possible explanation might be the previously reported secretion of YFV recombinant subviral particles (RSP) following prME expression by vaccinia vectors [20], [57]. This secreted particulate form of the antigen displays a surface structure similar to natural YFV virions resulting in improved antigenicity. In a previous study, DNA vaccines were compared that expressed either the complete TBE prME, or a truncated form producing soluble antigens that were unable to form particles. It was observed that protection by the particulate form was far superior to the soluble secreted form [58]. We speculate that the secretion of RSPs by vaccinia vectored vaccines might also contribute to the good protection levels observed in the current study.

A further advantage of using non-replicating poxviral vectors is the well described safety profile [31], [32], [36], [37], [44]. MVA is well tolerated in immune compromised [59], [60] and in elderly persons [61]. Using an *in vivo* safety assay, we showed that both vaccinia recombinants are safe, even at a dose of 10^7^ TCID_50_, when administered intracerebrally. In contrast, a much lower dose of 10^3^ TCID_50_, i.e. one tenth of the human dose of the YFV-17D killed 100% of the mice, using the same route. The superior safety of the vaccinia recombinants is probably due to the fact that the recombinants do not undergo a complete replication cycle while YFV-17D continues to propagate in the organism.

The effect of pre-existing antibodies to the vector in a part of the human population due to prior vaccinia vaccinations has been discussed extensively. Findings regarding the influence of anti-poxviral immunity have been contradictory [62]–[65]. In our hands pre-vaccination with a single dose of replicating vaccinia virus Lister or with wild-type MVA followed by prime/boost immunization with a suboptimal dose of MVA-YF had no measurable negative influence on protection. Even though protection in mice pre-immunized twice with wild-type MVA appeared diminished, the difference was not significant compared to non pre-treated mice. Nine months old mice used in the pre-immunization experiment revealed decreased survival rates (56% and 91%) as compared to nine weeks old mice (82% and 100%), which may be due to a less effective immune system in aged mice (see e.g. refs. [66]–[68]).

Protection studies in the present work were performed using intracerebral challenge with a fully lethal dose of a YFV vaccine strain, similar to earlier studies [20], [21]. Vaccine strains show virulence similar to wild-type strains, if administered intracerebrally [69]. The attenuated vaccine strain 17D was chosen for i.c. challenge for availability reasons and because of biosafety considerations. Animal models reflecting the viscerotropic character of the human disease have also been developed previously [23], [70], [71]. The assessment of the recombinant candidate vaccines in a viscerotropic model is desirable and will be a subject of further studies.

In summary, our data show that the non-replicating MVA-YF and dVV-YF candidate vaccines induce both cellular and humoral immune responses and protect mice from a multifold lethal challenge with YFV-17D, suggesting their usefulness as effective and safe live vaccines against yellow fever disease.
